# Minimally invasive posterior stabilization for treating spinal tuberculosis

**DOI:** 10.1007/s10195-012-0184-0

**Published:** 2012-02-23

**Authors:** S. Rigotti, L. Boriani, C. A. Luzi, S. Marocco, A. Angheben, A. Gasbarrini, C. Zorzi

**Affiliations:** 1 Ortopedia e Traumatologia, Ospedale Sacro Cuore Negrar Verona, Verona, Italy; 2 Malattie Tropicali, Ospedale Don Calabria Negrar Verona, Verona, Italy; 3 Chirurgia Vertebrale a Indirizzo Oncologico e Degenerativo Istituti Ortopedici Rizzoli Bologna, Bologna, Italy

**Keywords:** Spondylodiscitis, Spinal tuberculosis, Minimally invasive stabilization

## Abstract

We describe a case of dorsal–lumbar vertebral tuberculosis (Pott’s disease) first treated with antibiotic therapy, bed rest, and cast. After 2 months of treatment patient’s symptoms worsened. Minimally invasive posterior vertebral stabilization was carried out, with excellent clinic and radiographic results.

## Introduction

Surgical treatment of pyogenic and tubercular (TB) continues to evolve, thanks to minimally invasive techniques [5]. Standard indications to surgery are one or a combination of the following pathological settings: severe bone loss, progressively enlarging abscess, failure of conservative treatment, severe instability and/or deformity, or progressive neurologic deficit. A mainstay of orthopedic science has always been to avoid metallic implant positioning in an infected area. This case supports the safety of instrumentation in patients affected by pyogenic and TB vertebral osteomyelitis and its importance in the healing process by stabilizing the affected levels. The common trend is to widen the surgical indication [1] to achieve greater stability after extensive debridement of the infected area. In this context, a new treatment concept is to consider selected cases of spondylodiscitis as pathological fractures that need stabilization. The aim of this treatment is to create the best environment in which to allow antibiotics to work and heal the infection, making the new bone formation easier and filling the bone loss. We present a patient affected by Pott’s disease of T11–L1 treated successfully with antitubercular drugs and percutaneous sinal stabilization.

## Case report

**Fig. 1:**
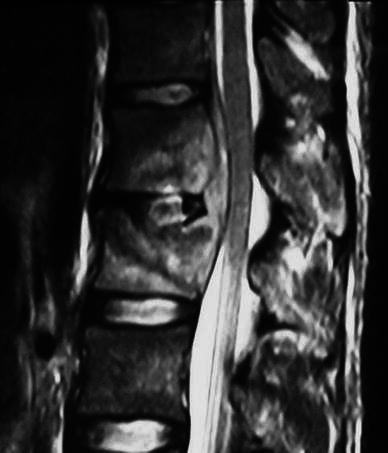
T2-weight magnetic resonance image, sagittal view. Involvement of T12 and L1 with progressive narrowing of the spinal canal

**Fig. 2:**
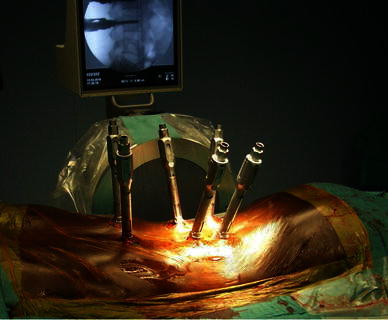
Intraoperative percutaneous posterior stabilization with C-arm control while preserving blood loss

Our patient is an African man 34 years old. He presented with back pain and malaise but no neurological deficit. He had been treated elsewhere with nonsteroidal anti-inflammatory drugs (NSAIDs); only after 24 weeks did he come to our practise. He was studied with standard X-ray, and magnetic resonance imaging (MRI) with gadolinium enhancement, which showed spondylodiscitis of T11-T12-L1 with psoas abscess and vertebral-canal stenosis. Clinical evaluation showed back pain on the visual analog scale (VAS) of 7/10 and no neurological deficit to the lower limbs; laboratory tests depicted high values of inflammation parameters, such as C-reactive protein (CRP) (28.98 mg/L; normal <5 mg/L). An X-ray-guided biopsy was conducted at T12-L1 under local anesthesia, and a *Mycobacterium tuberculosis* complex was isolated from the pus. Therefore, we started treatment with standard anti-TB treatment rifampin (RMP) 600 mg/day, isoniazid (INH) 300 mg/day, pyrazinamide (PZD) 1,500 mg/day, and ethambutol (ETB) 1,200 mg/day, bed rest, and a brace with weight bearing. After 2 months of therapy, the patient presented with weight loss, back pain (VAS 7/10, unmodified compared with the previous evaluation), serious asthenia (walking <50 meters), and no neurological deficit to the lower limbs. Laboratory tests showed decrease values of inflammation parameters, such as CPR (1.98 mg/L); MRI with gadolinium enhancement showed improved bone quality in T11, with partial, but not complete, infection resolution, narrowing of the right psoas abscess, and worse T12-L1 involvement (Fig. 1). On the basis of these results, we conducted a posterior percutaneous stabilization with bipedicular screws in T11-L2-L3 under general anesthesia, followed by standard X-ray. Thus, muscle tissue could be preserved and extensive blood loss avoided (Fig. 2). No postoperative restraint was applied, and the patient was put on his feet 2 days later without pain. The mean postoperative VAS improved to 2/10 2 weeks after surgery. One month after surgery, a computed tomography (CT) scan confirmed correct screw positioning and reestablishment of the sagittal axis. Clinically, the follow-up was characterized by restored body weight, and after having not having been permitted to ambulate, the patient was able to walk for more than 1,000 m without problems and returned to normal activities of daily life.

**Fig. 3:**
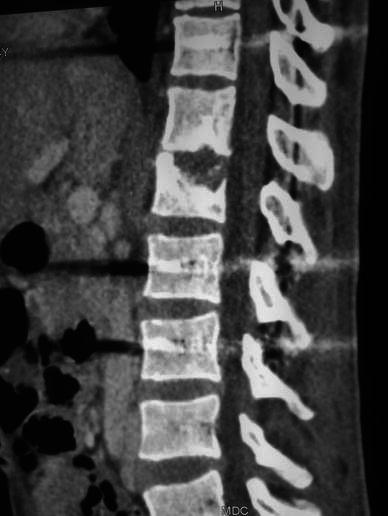
Computed tomography scan 12 months after surgery. Apposition of new bone tissue in T12, improved bone quality of L1, and maintenance of screws positioning

After the operation, our patient continued drug therapy with RMP and INH for 10 months. At 12 months, a CT scan (Fig. 3) showed sagittal axis maintenance, especially the apposition of new bone tissue.The patient provided his consent to publication of his case.

## Discussion

The goals of spondylitic TB treatment are care of the infection with adequate anti-TB drugs and care of the altered biomechanics of the functional segment involved (vertebra and intervertebral disc) through stabilization of the spine. Partial or complete resolution of pain and neurological deficits is the consequence of correct treatment. A multidisciplinary approach to these diseases is mandatory. We believe that antibiotic therapy is the appropriate initial treatment to cure the infection. However, spinal stabilization is another method for suppressing spinal infections and preventing serious imbalance in kyphosis that can cause severe neurological impairment [2], especially after injury to the thoracic spine. In that case, the sagittal weight-bearing axis is anterior to the center of the vertebral bodies and produces progressive kyphotic deformation [3]. Stabilization of a bony lesion is a well-known and important factor in the healing process of infections, creating the optimal environment for antibiotics to exert their effect in the area. The use of metallic implants in an infected area of the spine is safe and does not lead to persistence or recurrence of infection if associated with correct antimicrobial therapy [1]. Several techniques describe using an anterior and posterior approach in patients with no neurologic disorder, and percutaneous internal fixation is useful as a minimally invasive approach to reduce immobilization and recovery time in fragile patients [4]. Our patient had a good response to drug therapy but nonetheless displayed increasing weakness, fatigue, and weight loss. Only after restored spinal stability did his response to antibiotic therapy improve, with improvement in pain and functional activities, which prevented neurological deficits. Comparison of follow-up imaging showed correction stability and healing of diseased tissue, along with new bone apposition. Minimally invasive techniques preserve muscle tissue and obtain spinal stabilization with minimal blood loss, thereby allowing a rapid recovery, which is essential in vulnerable patients. A single case cannot demonstrate the effectiveness and safety of a surgical technique, so only with clinical trials will it be possible to draw ultimate conclusions.
